# Editorial: Impact of female hormones on the brain

**DOI:** 10.3389/fendo.2024.1451286

**Published:** 2024-08-14

**Authors:** Jean-Michel Le Melledo, Caroline Gurvich, Jayashri Kulkarni

**Affiliations:** ^1^ Department of Psychiatry, Faculty of Medicine & Dentistry, University of Alberta, Edmonton, AB, Canada; ^2^ Department of Psychiatry, Faculty of Medicine, Nursing & Health Sciences, Monash University, Melbourne, VIC, Australia

**Keywords:** estrogens, depression, female, brain, HPA axis

The specificities of women, including women’s brains, have been neglected in research over many years. Discussions about the inclusion of women in research only became topical in the 1970s, as part of the women’s health movement. However, in 1977, a Food and Drug Administration policy recommended excluding women of childbearing potential from Phase I and early Phase II drug trials (1). This policy was a reaction to adverse drug-related incidents in pregnant women who had participated in clinical trials; however, the implications of this led to a shortage of data on how many drugs affect women, as well as broader gap in knowledge about neuroscience related to sex and gender. In response to this knowledge gap, NIH developed a policy on the inclusion of women in clinical research, which became law in 1993. Since that time, the importance of reporting of research results with specification of both biological sex and gender has been recognised (2). The more specific impacts of female hormones on the brain has only attracted interest in recent decades and investigations on this topic are leading to dramatic advances in clinical understanding and applications.

Hantsoo et al. elegantly reviewed female-specific mood disorders such as premenstrual dysphoric disorder, postpartum depression and perimenopausal depression. They stressed the tremendous interplay, especially in women with a history of trauma, between the hypothalamic-pituitary-adrenal (HPA) axis and female hormones in the occurrence of depression across the female reproductive lifecycle. Interestingly, Hantsoo et al. suggest that studies comparing HPA axis function between females with premenstrual dysphoric disorder with and without a history of trauma are needed. This is the objective of the investigation by Nayman et al.. This research team demonstrated that women with premenstrual dysphoric disorder and a history of childhood trauma exhibited lower cortisol levels during the luteal phase of the menstrual cycle compared to the follicular phase of the menstrual cycle whereas no such cyclicity was observed with women with premenstrual dysphoric disorder without such a history. These results illustrate substantial heterogeneity in the physiopathology of premenstrual dysphoric disorder.

In their research on the role of endogenous and exogenous sex hormones (i.e. oral contraceptives) on morphologic alterations of the fear circuitry Brouillard et al. found that both endogenous and exogenous sex hormones can affect brain morphology. They demonstrated sex differences in grey matter volume in brain regions associated with fear circuitry, as well as reporting that combined oral contraceptives can affect fear-related brain morphology but noted that the effects of exogenous sex hormones were reversible over time. Smith and Smilek suggest a potential positive effect of oral contraceptives in undergraduate female students, with a potential increase in self-regulatory actions in goal pursuit. The authors, however, acknowledge possible limitations related to their sample selection. Both Brouillard et al. and Smith and Smilek recognize the complexity of assessing the effects of hormonal contraceptives, especially considering the large diversity of hormonal formulations available.

Further research in the effects of exogenous administration of female hormones is critical for the development of new hormonal therapeutics for use in neuropsychiatric disorders, neurodevelopmental disorders and aging.

However, such research cannot be performed without a very thorough understanding of the multiple and complex changes produced by these female hormones and their effects on the brain. Bendis et al. offer a detailed review of the multiple mechanisms of action of estrogens on neurotransmitters (serotonin, dopamine, glutamate), dysregulations of which contribute the physiopathology of multiple neuropsychiatric disorders. The perimenopause is associated with fluctuating and decreasing levels of estrogens including in the hypothalamus, which triggers vasomotor symptoms such as hot flushes and night sweats as well as being directly associated with an increased risk of depression. Prokai-Tatrai and Proka provide a detailed review on the effects of declining levels of estradiol on the brain and in particular how estrogen deficiency is related to vasomotor symptoms. Based on recent greater understanding of the effects of estrogens on the brain, they are hopeful that a pro-drug they developed (DHED) will selectively convert to estrogen in the brain, without peripheral negative effects and therefore will treat vasomotor symptoms of perimenopause/menopause without adverse effects.

The higher prevalence and worse course of illness observed in women with Alzheimer’s disease make the research of Onisiforou et al. highly relevant. Using transcriptomic analysis, they demonstrated differences in the regulation of the estrogen receptor alpha gene in the hippocampus of males and females suffering from Alzheimer’s disease. Furthermore, they report an upregulation of the estrogen signalling pathway in male patients with Alzheimer’s disease. Sakaguchi and Tawata, as other authors of this Research Topic, underline the importance of investigating the brain hormonal milieu, and particularly female hormones, in the context of atypical sex differentiation. In their thorough review of the literature regarding giftedness and atypical sexual differentiation, they suggest that insufficiency of estrogen receptor beta functions in neural cell development is critical in the emergence of autism spectrum conditions.

The best example of the tremendous need for investigating women specificities and the potential clinical benefits for all is illustrated by the research on allopregnanolone, a neuroactive steroid which is a metabolite of progesterone. It was first demonstrated to be a potent anxiolytic in animal models and then shown to be a very potent endogenous allosteric modulator of the GABA-A receptor with an ability to change the subunit composition of the GABA -A receptor and its pharmacological properties. Years later, an analogue of allopregnanolone was the first medication approved by the Food and Drug Administration (FDA) for the treatment of postpartum depression (3). Another analogue of allopregnanolone was recently found to be a rapid and effective antidepressant in women and men (4).
